# LncRNA CRNDE promotes the epithelial‐mesenchymal transition of hepatocellular carcinoma cells via enhancing the Wnt/β‐catenin signaling pathway

**DOI:** 10.1002/jcb.26762

**Published:** 2018-11-14

**Authors:** Liying Zhu, Nenghong Yang, Guiqin Du, Chengcheng Li, Guoqi Liu, Shengju Liu, Yongjie Xu, Yanan Di, Wei Pan, Xing Li

**Affiliations:** ^1^ Department of Medical Laboratory Affiliated Hospital of Guizhou Medical University Guiyang Guizhou China; ^2^ Department of Hepatobiliary Surgery Surgery, Affiliated Hospital of Guizhou Medical University Guiyang China; ^3^ The First People's Hospital of Guiyang Guiyang Guizhou China; ^4^ Department of Clinical Laboratory Medicine Beifang Hospital of China North Industries Group Corporation Beijing China

**Keywords:** colorectal neoplasia differentially expressed (CRNDE), epithelial‐mesenchymal transition (EMT), hepatocellular carcinoma (HCC), Wnt/β‐catenin signaling pathway

## Abstract

Colorectal neoplasia differentially expressed (CRNDE) is a significantly upregulated long noncoding RNA in hepatocellular carcinoma (HCC). CRNDE could promote cell proliferation, migration, and invasion, while its molecular mechanisms were still largely unclear. In this study, we investigated the expression and function of CRNDE. CRNDE was significantly upregulated in tumor tissues compared with adjacent normal tissues. In vitro, we revealed that knockdown of CRNDE inhibited cell proliferation, migration, and cell invasion capacities in HCC. Animal studies indicated that CRNDE knockdown represses both growth and metastasis of HCC tumors in vivo. Moreover, knockdown of CRNDE suppressed the cell epithelial‐mesenchymal transition (EMT) process by increasing the expression of E‐cadherin and ZO‐1, whereas, decreasing the expression of N‐cadherin, slug, twist, and vimentin in HCC cells. We also revealed that knockdown of CRNDE suppressed the Wnt/β‐catenin signaling in HCC. Thus, CRNDE could modulate EMT of HCC cells and knockdown of CRNDE impaired the mesenchymal properties. CRNDE increased invasion of HCC cells might be through activating the Wnt/β‐catenin signaling pathway.

## 1 INTRODUCTION

Hepatocellular carcinoma (HCC) is the most prevalent primary liver cancer worldwide. Metastases with HCC have an extremely poor prognosis.^1^ Thus, to explore the new target for HCC treatment is important.

Long noncoding RNAs (lncRNAs) are a group of non–protein coding RNAs that are greater than 200 nucleotides in length. Numerous studies have demonstrated that lncRNAs are often dysregulated in HCC. LncRNAs are likely to be involved in diverse biological processes, such as cell proliferation, apoptosis, epithelial‐mesenchymal transition (EMT), invasion and metastasis, and so on. LncRNA LINC01186, regulated by TGF‐β/SMAD3, inhibits cell migration and invasion through EMT in lung cancer.^2^ LncRNA UCA1 promotes EMT of breast cancer cells by enhancing the Wnt/β‐catenin signaling pathway.^3^ H19 and miR‐675 play crucial roles in metastasis, through the regulation of critical events specifically the epithelial to mesenchymal and the mesenchymal to epithelial transitions.^4^ LncRNA FEZF1‐AS1 enhances EMT through suppressing E‐cadherin and regulating the Wnt pathway in non–small cell lung cancer.^5^

The Wnt/β‐catenin pathway is a canonical Wnt‐signaling pathway that regulates several biological processes, including proliferation, apoptosis, migration, and invasion.^6^ Abnormal activation of the Wnt/β‐catenin signaling pathway may serve an important role in the pathogenesis of various human diseases, particularly in human cancer.

LncRNA colorectal neoplasia differentially expressed (CRNDE) is activated early in colorectal neoplasia.^7^ Recent reports have demonstrated that CRNDE regulates cell proliferation, apoptosis, migration, and invasion in various cancers.^8^, ^9^, ^10^ But the underlying mechanism of CRNDE regulating EMT in HCC is still largely unclear. Herein, we demonstrated that lncRNA CRNDE was upregulated in HCC tissues compared with adjacent normal tissue. In vitro, inhibition of CRNDE inhibited cell proliferation, cell invasion, and EMT process in HCC. In vivo, inhibition of CRNDE repressed tumor growth and metastasis in HCC. We also indicated that CRNDE modulated Wnt/β‐catenin signaling in HCC cells. Thus, CRNDE may be a potential target of HCC treatment.

## 2 MATERIALS AND METHODS

### 2.1 HCC tissue samples and cells culture

The 12 pairs of tumor and adjacent nontumor tissues were from surgical resections of HCC in the Affiliated Hospital of Guizhou Medical University (Guizhou, China). Specimens were snap‐frozen in liquid nitrogen and stored at −80°C. All of the patients received surgery, and without preoperative chemotherapy or radiation therapy. All patients provided written informed consent and ethical consent was granted from the Committees for Ethical Review of Research involving the Affiliated Hospital of Guizhou Medical University.

Human HCC cell lines (HepG2, SMMC7721, SK‐hep1, and Huh7), human immortalized, normal liver cell line (L02), and the embryonic kidney cell line 293T were obtained from the Chinese Academy of Sciences Cell Bank. They were cultured in the Dulbecco modified Eagle medium of high glucose with 10% fetal bovine serum (FBS; BI, ISR). All cells were incubated at 37°C in a humidified atmosphere with 5% CO_2_.

### 2.2 RNA extraction and quantitative real‐time polymerase chain reaction

Total RNA was isolated with TRIzol reagent (Invitrogen, Carlsbad, CA) and treated with RNase‐free DNase (Promega, Madison, WI). For CRNDE, the first‐strand complementary DNA was generated using the PrimeScript RT reagent kit with gDNA Eraser according to the manufacturer's instructions (Takara, Dalian, China). The primers used were 5′‐ATATTCAGCCGTTGGTCTTTGA‐3′ (forward) and 5′‐TCTGCGTGACAACTGAGGATTT‐3′ (reverse). GAPDH was used for normalization, the primers for it were 5′‐CGACCACTTTGTCAAGCTCA‐3′ (forward), 5′‐AGGGGTCTACATGGCAACTG‐3′ (reverse). All quantitative real‐time polymerase chain reaction (qRT‐PCR) samples were performed by using the FastStart Universal SYBR Green Master mixture (Roche, Basel, Switzerland and lowa, IL). The quantification analysis was analyzed by the $2^{-\Delta \Delta C_{t}}$ method.

### 2.3 Construction of short‐hairpin RNA and transfection

The short‐hairpin RNA (shRNA) targeting human CRNDE were ligated into the pGreenPuro shRNA vector (SBI, Palo Alto, CA) according to the manufacturer's protocol. Transfections were performed with a Lipofectamine 2000 kit (Invitrogen) according to the manufacturer's instructions. The cells were harvested 48 to 72 hours after transfection.

### 2.4 MTS assay

Cells (2000 cells/well) were seeded into 96‐well plates after 24 hours of transfection, and measured at different time points (0, 24, 48, and 72 hours) using the MTS kit (CellTiter 96 Aqueous One Solution Cell Proliferation Assay, Promega), followed the manufacturer's protocol. The 490 nm wavelength absorption value was measured. All experiments were performed in triplicate and repeated three times.

### 2.5 Wound‐healing assay

Cells were plated at a density of 1 × 10^5^ cells/well in 6‐well plates and incubated at 37°C. Once cells were attached completely, the medium was replaced with serum‐free medium. A wound was produced by scraping across the cell monolayer using a 200‐μL sterile polystyrene micropipette tip. After incubation for 48 hours, we measured the fraction of cell coverage across the line.

### 2.6 Cell migration and invasion assay

Twenty‐four well chambers with 8 μm pore size (Costar 3422; Corning, NY) were used in cell migration and invasion assays. Cells (5 × 10^4^) in 100 μL of serum‐free media were seeded into the upper chamber (without or precoated with 500 ng/mL Matrigel solution [BD, Franklin Lakes, NJ] in migration or invasion assay separately); 600 μL of 10% FBS medium was placed in the lower chamber. After 48 hours of incubation, the upper chambers were removed from the plates and cells on the top side of the chamber were wiped with a cotton swab. Migrating or invading cells were fixed and then stained by Giemsa staining. Cells were counted under a microscope (Olympus, Tokyo, Japan). Five randomly fields were counted under a microscope and photos were taken.

### 2.7 Western blot analysis

Cells were lysed in RIPA buffer (Beyotime, Shanghai, China), supplementing with 1 mmol/L PMSF, and protein concentration was measured by the BCA Assay Kit (Beyotime). Proteins were separated on a 10% to 12% SDS‐PAGE gel and transferred to a PVDF membrane. The membrane was blocked with 5% milk, incubated overnight at 4°C with a primary rabbit antibody. Antibody dilutions of 1:500 were used for E‐cadherin (Bioword, Nanjing, China), ZO‐1 (Proteintech, Wuhan, China), N‐cadherin (Bioword), slug (Bioword), twist (Bioword), vimentin (Bioword), Wnt2 (Bioword), Frizzled 4 (Bioword), β‐catenin (Bioword), and 1:2000 for GAPDH (Proteintech). The secondary antibodies applied a goat anti‐rabbit or anti‐mouse horseradish peroxidase (HRP) IgG (1:2000; Bioword). ECL Detection Reagent (Millipore, Billerica, MA) was used to detect the signal.

### 2.8 Immunohistochemistry

Immunohistochemistry for Ki‐67 was performed on paraffin sections using a primary antibody against Ki‐67 (Proteintech) and an HRP‐conjugated rabbit anti‐goat antibody (Maixin, Fuzhou, China), and the proteins in situ were visualized with 3,3‐diaminobenzidine and analyzed using a bright field microscope.

### 2.9 Immunofluorescence analysis

Different treated HCC cells were cultured and fixed on 14 × 14 mm glass slides. After first incubated with antibodies specific for E‐cadherin (Bioword), ZO‐1 (Proteintech), N‐cadherin (Bioword), slug (Bioword), twist (Bioword), vimentin (Bioword) and then with goat anti‐rabbit IgG (Alexa Fluor 488; Proteintech), the slides were mounted by adding DAPI Fluoromount‐G (Beyotime) and examined with an Eclipse Ti‐S inverted phase/fluorescent microscope (Nikon, Tochigi, Japan).

### 2.10 Construction of stable cell lines

To obtain cell lines stably interfering CRNDE, the shRNA targeting human CRNDE were ligated into the pGreenPuro shRNA vector (SBI) according to the manufacturer's protocol. Transfected SMMC7721 cells were selected with puromycin (1 µg/mL) for 4 weeks. Selected cells were further subcloned for uniform stable cell lines. The stably interfering cell lines were identified using qRT‐PCR.

### 2.11 Animal studies

The male BALB/c nude mice (4‐6 weeks old) were purchased from the Laboratory Animal Services Center of Guizhou Medical University. Ten mice were randomly allocated into two groups. A total of 1 × 10^7^ SMMC7721 cells stably interfering CRNDE (sh‐CRNDE) and control cells were subcutaneously into the dorsal flanks of mice. Tumor growth was examined at the indicated time points and tumor volumes were measured by using the equation *V* (mm^3^) = *A* × *B* ^2^/2, where *A* is the largest diameter and *B* is the perpendicular diameter. After 28 days, the mice were killed and tumors were removed and weighed.

SMMC7721 cells stably interfering CRNDE or negative control were propagated and 1 × 10^7^ cells were injected into the liver tissue of mice, respectively. After 6 weeks, mice were killed for intrahepatic metastasis assessment.

### 2.12 Statistical analysis

Statistical analyses were performed using SPSS 17.0 software (SPSS, Chicago, IL). Differences between two groups were assessed using the Student *t* test (two‐tailed). Each experiment was performed at least three times. Results of experiments are displayed as a mean ± standard deviation. A *P* value < 0.05 was considered to indicate statistical significance.

## 3 RESULTS

### 3.1 CRNDE was upregulated in HCC

**Figure 1:**
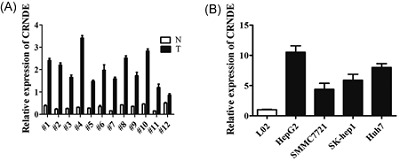
CRNDE was significantly upregulated in HCC tissues and cell lines. A, CRNDE expression level was examined in 12 paired HCC tissues (T) and nontumor tissues (N) by qRT‐PCR. Transcript levels were normalized to GAPDH expression. B, CRNDE expression level was examined in HCC cell lines (HepG2, SMMC7721, SK‐hep1, and Huh7) and one normal liver cell line L02 by qRT‐PCR. Transcript levels were normalized to GAPDH expression. CRNDE, colorectal neoplasia differentially expressed; HCC, hepatocellular carcinoma; qRT‐PCR, quantitative real‐time polymerase chain reaction

We evaluated the CRNDE expression level in 12 paired HCC and adjacent normal tissue samples using reverse transcription and qRT‐PCR. The results showed that CRNDE levels were significantly higher in tumor tissues (Figure 1A). We then examined the expression level of CRNDE in HCC cell lines (HepG2, SMMC7721, SK‐hep1, and Huh7) and human immortalized, normal liver cell line (L02). Compared with L02, cells exhibited significantly higher levels of CRNDE expression (Figure 1B).

### 3.2 Knockdown of CRNDE inhibited HCC cell growth, migration, and invasion in vitro

**Figure 2:**
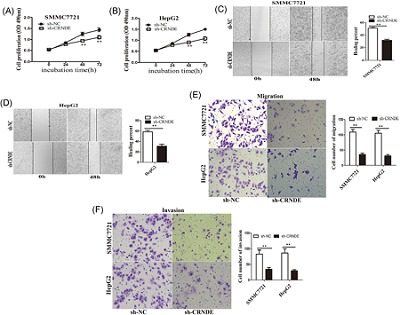
Knockdown of CRNDE inhibited HCC cell proliferation, migration, and invasion in vitro. A,B, MTS cell proliferation was detected after cells were transfected with sh‐NC, sh‐CRNDE at 0, 24, 48, and 72 hour in SMCC7721 and HepG2 cells. Representative graphs are shown. C,D, Wound‐healing assay for determining the effect of CRNDE knockdown on the healing of SMMC7721 and HepG2 cells. Representative graphs are shown. ^**^ *P* < 0.01. E,F, Cell migration and invasion assay was examined and cell number was calculated after cells were transfected with sh‐NC, sh‐CRNDE at 24 hour in SMCC7721 and HepG2 cells. Data are shown as mean ± SD (n = 3) and are representative of three independent experiments.^**^ *P* < 0.01. CRNDE, colorectal neoplasia differentially expressed; HCC, hepatocellular carcinoma; qRT‐PCR, quantitative real‐time polymerase chain reaction; SD, standard deviation

To evaluate the possible role of CRNDE in HCC, we transfected HCC cell lines with interfering CRNDE (sh‐CRNDE). The growth curves detected by MTS showed that CRNDE knockdown significantly decreased HCC cell growth (Figure 2A and 2B). Knockdown of CRNDE also suppressed HCC cell migration an invasion. In wound‐healing assays, CRNDE knockdown decreased migratory speed (Figure 2C and 2D). Transwell chamber assays confirmed that decreased cell migration after CRNDE knockdown in both SMMC7721 and HepG2 cell lines (Figure 2E and 2F). As the transwell chamber assay could only verify changes in the migratory capacity of HCC cells, we investigated invasive ability alterations using Matrigel‐coated transwell experiments and found fewer cells invading through when knockdown of CRNDE (Figure 2E and 2F).

### 3.3 Knockdown of CRNDE also repressed HCC growth and metastasis in vivo

**Figure 3:**
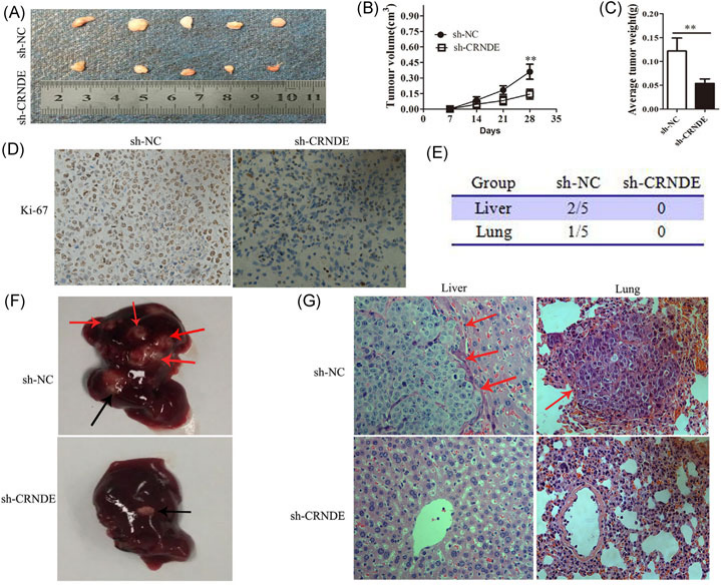
CRNDE knockdown also inhibited HCC growth and metastasis in vivo. A‐D, Subcutaneous tumor model of CRNDE knockdown SMMC7721 cells (n = 5 for both groups). A, The subcutaneous tumor model of stable CRNDE‐interference SMMC7721 cells (n = 5 for both groups). B,C, Tumor volume and weight at the endpoint. D, The tumor sections were under IHC staining using antibodies against Ki‐67, the representative graph are shown in #1 sample. Magnification is ×400. E‐G, The orthotopic xenograft model of CRNDE‐interference SMMC7721 cells (n = 5 for both groups). E, Incidence of metastases 6 weeks after orthotopic implantation. Representative photographs of livers. Black arrow: implanted orthotopic tumors; red arrows: metastatic foci. G, Representative histological image of H&E staining. Red arrows: metastatic foci. Magnification is ×400. CRNDE, colorectal neoplasia differentially expressed; HCC, hepatocellular carcinoma; qRT‐PCR, quantitative real‐time polymerase chain reaction

To further probe the effects of CRNDE in vivo, sh‐CRNDE and controlled (sh‐NC) SMMC7721 cells were injected subcutaneously into the dorsal flanks of mice. Compared with those produced from control xenografts, tumor growth was slowed by knockdown of CRNDE (Figure 3A). Furthermore, the final tumor volume and weight were smaller in the sh‐CRNDE group than in the control group (Figure 3B and 3C). Moreover, the immunohistochemistry analysis of the tumor tissues revealed that the expression of Ki‐67 proliferation antigen was significantly weaker in the sh‐CRNDE group than the sh‐NC group (Figure 3D).

Next, we tested HCC cell invasive behavior changes in orthotopic tumor models. We found no metastatic nodules in the livers of mice in the sh‐CRNDE group (0/5) compared with the control group (2/5) (Figure 3E and 3F). Moreover, knockdown of CRNDE also repressed lung metastases of SMMC7721 cells (Figure 3E). Histopathological analysis by H&E staining confirmed liver and lung metastatic foci by the control cells (Figure 3G). Taken together, these data demonstrated that knockdown of CRNDE represses both growth and metastasis of HCC tumors in vivo.

### 3.4 Knockdown of CRNDE suppressed the cell EMT process in HCC

**Figure 4:**
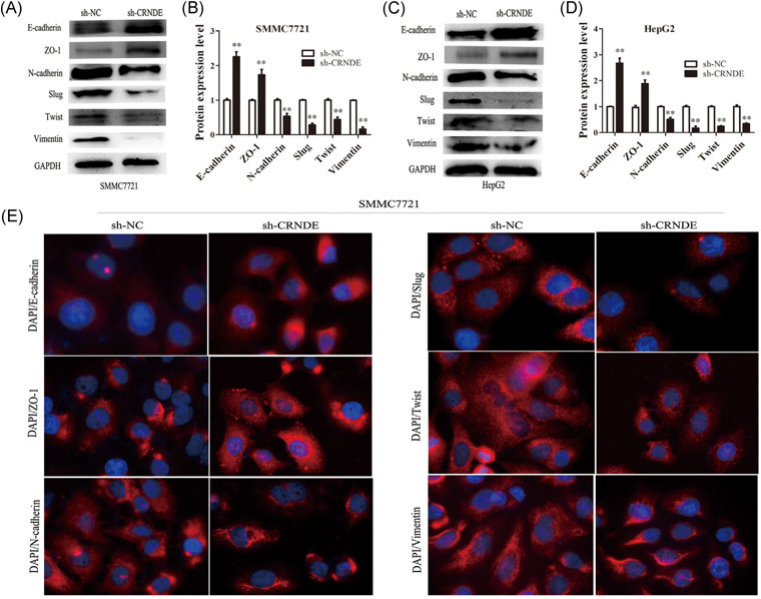
Knockdown of CRNDE suppressed cell EMT process in HCC. A‐D, The relative expression levels of E‐cadherin, ZO‐1, N‐cadherin, slug, twist, and vimentin were showed after knockdown of CRNDE in SMMC7721 and HepG2 cells. Each data represents the mean ± SD, ^**^ *P* < 0.01. E, Representative images of immunofluorescence staining of E‐cadherin, ZO‐1, N‐cadherin, slug, twist, and vimentin in SMMC7721 cells with or without CRNDE inhibition. Blue: DAPI. Red: The antibody for E‐cadherin, ZO‐1, N‐cadherin, slug, twist, and vimentin. CRNDE, colorectal neoplasia differentially expressed; EMT, epithelial‐mesenchymal transition; HCC, hepatocellular carcinoma; qRT‐PCR, quantitative real‐time polymerase chain reaction

It is clear that EMT is involved in metastatic events in cancer. Therefore, we further investigated whether CRNDE can regulate EMT of HCC cells. Western blot analysis showed that CRNDE knockdown had significantly upregulated the expression of epithelial markers E‐cadherin and ZO‐1, whereas, reduced the expression levels of transcription factors slug, twist, and mesenchymal markers N‐cadherin and vimentin in SMMC7721 and HepG2 cells (Figure 4A‐D). To further verify these changes, we performed immunofluorescence analysis. The results confirmed that CRNDE knockdown resulted in increased expression of E‐cadherin and ZO‐1, whereas, reduced expression of a slug, twist, N‐cadherin, and vimentin in SMMC7721 cells (Figure 4E). Taken together, these results showed that knockdown of CRNDE impaired the mesenchymal properties in HCC cells.

### 3.5 CRNDE modulated Wnt/β‐catenin signaling in HCC cells

**Figure 5:**
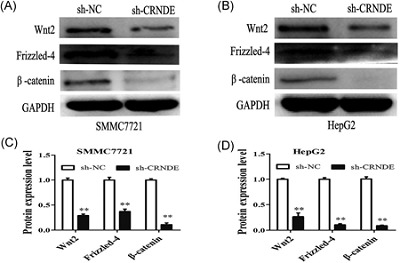
CRNDE modulated Wnt/β‐catenin signaling in HCC cells. A,B, Western blot analysis of Wnt2, Frizzled 4, and β‐catenin were showed after knockdown of CRNDE. C,D, Measurement of the relative protein expression of Wnt2, Frizzled 4, and β‐catenin. Each data represents the mean ± SD, ^**^ *P* < 0.01. CRNDE, colorectal neoplasia differentially expressed; HCC, hepatocellular carcinoma; SD, standard deviation

The canonical Wnt/β‐catenin signaling pathway plays an important role in EMT and metastasis.^11^, ^12^ The expression of Wnt2 is implicated in activating/stabilizing β‐catenin, similar to other canonical (β‐catenin‐mediated) Wnt ligands.^13^, ^14^, ^15^ Frizzled 4, an essential receptor in the Wnt/β‐catenin signaling. The nuclear translocation of β‐catenin results in the activation of canonical Wnt/β‐catenin signaling, leading to the transcriptional activation of downstream targets.^16^ Thus, we further examined whether CRNDE affected the Wnt/β‐catenin signaling. Western blot analysis showed that knockdown of CRNDE significantly suppressed the protein expression levels of Wnt2, Frizzled 4, and β‐catenin (Figure 5A‐D). These data indicated that CRNDE knockdown inhibits Wnt/β‐catenin activation.

## 4 DISCUSSION

Numerous studies have demonstrated that CRNDE is upregulated and promote proliferation and metastasis in various cancers, including colorectal cancer, gastric cancer, glioma, non–small cell lung carcinoma, and hepatic carcinoma.^8^, ^9^, ^17^, ^18^, ^19^ In the study, our finding demonstrated that CRNDE is upregulated in HCC tissues compared with adjacent normal tissues. Function assays showed that knockdown of CRNDE suppresses cell proliferation, migration, and cell invasion abilities in vitro. Animal studies indicated that CRNDE knockdown represses both growth and metastasis of HCC tumors in vivo.

EMT is believed to be a major contributing mechanism to cancer invasion and metastasis.^20^, ^21^ Low expression of miR‐448 induces EMT and promotes invasion by regulating ROCK2 in HCC.^22^ Serine protease inhibitor Kazal type 1 downregulates E‐cadherin and induces EMT of hepatoma cells to promote HCC metastasis via the MEK/ERK signaling pathway.^23^ Small nucleolar RNA 47 promotes tumorigenesis by regulating EMT markers in HCC.^24^ MiR‐93 enhances HCC invasion and metastasis by EMT via targeting PDCD4.^25^ In the study, we demonstrated that CRNDE knockdown suppresses cell EMT process by downregulation of a slug, twist, N‐cadherin, and vimentin, whereas, upregulates the E‐cadherin and ZO‐1 expression levels in HCC cells.

Activation of the Wnt/β‐catenin signaling is one of the most important inducers of EMT.^26^ GSK‐3beta suppresses HCC cell dissociation in vitro by upregulating epithelial junction proteins and inhibiting the Wnt/β‐catenin signaling pathway.^27^ A novel oncolytic adenovirus targeting Wnt signaling effectively inhibits cancer‐stem like cell growth via metastasis, apoptosis, and autophagy in HCC models.^12^ Agkihpin, a novel SVAE may inhibit the migration and invasion of liver cancer cells associated with the inversion of EMT induced by the Wnt/β‐catenin signaling inhibition.^28^ Wnt ligands bind to Frizzled receptors and low‐density lipoprotein receptor‐related protein 5/6 coreceptors, which stabilizes β‐catenin protein by inhibiting the protein destruction complex composed of adenomatous polyposis coli, Axin1, casein kinase 1, and glycogen synthase kinase 3.^29^ Wnt2, a member of the WNT gene family, directs cell specification during development. Wnt2 activates canonical Wnt signaling and upregulates β‐catenin target genes.^30^, ^31^ Wnt2 expression is associated with anchorage‐independent cell survival, tumor invasion and metastasis in cancer cells.^32^, ^33^ Hepatitis C virus core protein upregulates gene expression of canonical Wnt ligands Wnt2 in the SMMC7721 cell line.^34^ WNT2 messenger RNA is upregulated in colorectal polyps, primary colorectal cancer of stage A‐C, and also in liver metastasis from colorectal cancer.^35^ Frizzled 4 is an essential receptor in the Wnt/β‐catenin signaling. The nuclear translocation of β‐catenin results in the activation of canonical Wnt/β‐catenin signaling, leading to the transcriptional activation of downstream targets.^16^ In the study, we demonstrated that knockdown of CRNDE downregulated Wnt2, Frizzled 4, and β‐catenin expression in HCC cells. The results indicated that CRNDE modulates the Wnt/β‐catenin signaling pathway in HCC.

In conclusion, our results showed that CRNDE was upregulated in HCC tissues. Knockdown of CRNDE repressed cell proliferation, migration, and invasion. We found that knockdown of CRNDE suppresses HCC cell invasion and metastasis through impairing the mesenchymal properties. Moreover, knockdown of CRNDE suppressed the Wnt/β‐catenin signaling in HCC. On the basis of these findings, we infer that CRNDE increases invasion of HCC cells via activating the Wnt/β‐catenin signaling pathway.
